# Monitoring the outcomes of non‐pharmacological treatments for cognitive impairment using magnetoencephalography: A case series

**DOI:** 10.1002/ccr3.8385

**Published:** 2023-12-28

**Authors:** Yoko Hirata, Hideyuki Hoshi, Momoko Kobayashi, Keita Shibamiya, Keisuke Fukasawa, Sayuri Ichikawa, Yoshihito Shigihara

**Affiliations:** ^1^ Department of Neurosurgery Kumagaya General Hospital Kumagaya Japan; ^2^ Precision Medicine Centre Hokuto Hospital Obihiro Japan; ^3^ Precision Medicine Centre Kumagaya General Hospital Kumagaya Japan; ^4^ Clinical laboratory Kumagaya General Hospital Kumagaya Japan

**Keywords:** cognitive dysfunction, magnetoencephalography, neuropsychological test, rehabilitation

## Abstract

**Key Clinical Message:**

Cognitive impairment associated dementia is treatable non‐pharmacologically. Monitoring tools are important to provide proper treatment. The present study showed that the resting‐state brain activity measured using magnetoencephalography reflects their outcomes and captures clinical impressions better than neuropsychological assessments, which have inherent limitations such as the practice effect.

**Abstract:**

Mild cognitive impairment (MCI) is a prodromal phase of dementia caused by brain diseases. Non‐pharmacological treatments are sometimes effective in improving patient's cognition and quality of life. To provide better treatments, monitoring the treatment outcomes, which is done using neuropsychological assessments, is important. However, these assessments have inherent limitations, such as practice effects. Therefore, complementary assessments are anticipated. Magnetoencephalography (MEG) is a neuroimaging technique that is sensitive to changes in brain activity associated with cognitive impairment. It represents the state of brain activity in terms of MEG spectral parameters associated with neuropsychological assessment scores. MEG spectral parameters could reasonably be used to monitor treatment outcomes without the aforementioned limitations. However, few published longitudinal reports have assessed MEG spectral parameters during the non‐pharmacological treatment period for cognitive impairment associated with dementia. In this study, we retrospectively examined the clinical records of two patients with MCI. Changes in neuropsychological assessment scores and MEG spectral parameters were qualitatively evaluated along with the patients' conditions, as described in the medical records during non‐pharmacological treatments provided for more than 2 years. The changes in neuropsychological assessment scores and MEG spectral parameters showed comparable trends, with some discrepancies. Changes in MEG spectral parameters were more consistent with the subjective reports from caregivers and medical staff in the medical records. Our results suggest that MEG is a promising tool for monitoring patient conditions during treatment.

## Graphical abstract

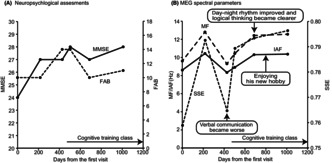

## 1 INTRODUCTION

Dementia and mild cognitive impairment (MCI) are syndromes characterized by cognitive deterioration due to brain diseases, such as Alzheimer's disease. Although only few effective drugs are available to treat the underlying diseases, non‐pharmacological treatments can also improve symptoms, including cognition and quality of life.^1^, ^2^ Treatment outcomes should be monitored using repeated assessments to provide effective treatment.

Cognitive status is typically assessed using neuropsychological assessments such as the Mini Mental State Examination (MMSE) and Frontal Assessment Battery (FAB).^3^ The MMSE assesses global cognitive function with an emphasis on learning and memory, whereas the FAB assesses executive functions. Even though neuropsychological assessments are well‐validated, they are unsuitable for repeated use, which leads to intrinsic problems such as practice effects.^4^, ^5^ These issues can seriously impact test quality, particularly when assessing patients with MCI, whose cognition is relatively preserved.

Magnetoencephalography (MEG) has been recently used in clinical practice in memory clinics to compensate for the limitations of neuropsychological assessments.^3^ This method evaluates resting‐state brain activity in terms of spectral parameters such as median frequency (MF), individual alpha frequency (IAF), and Shannon's spectral entropy (SSE).^6^ While the IAF has been positively correlated with the MMSE score, SSE has been positively correlated with the FAB score, and MF has been positively associated with both functions.^3^ Because lower scores on neuropsychological assessments indicate more severe cognitive impairment, lower values of spectral parameters also indicate more severe symptoms; these scores were lower in patients with MCI^7^ and dementia^6^ compared with healthy individuals. MEG spectral parameters are calculated from resting‐state MEG data without individuals performing any tasks and are not likely to be affected by repeated use (i.e., repeated measurements); they are thus suitable for monitoring the cognitive status of patients during the non‐pharmacological treatment period. Given that they capture the severity of cognitive impairments, these parameters are expected to increase when the patient's condition improves during non‐pharmacological treatments. However, no published longitudinal studies have reported changes in these parameters during non‐pharmacological treatments for patients with cognitive impairment associated with dementia. In the present study, we examined the clinical records of two patients with MCI who visited our memory clinic and participated in a weekly cognitive training class for more than 2 years. To determine whether the changes in MEG spectral parameters of these patients reflect their clinical changes, we qualitatively evaluated their medical records.

## 2 CASE REPORTS

### 2.1 Case 1

**FIGURE 1:**
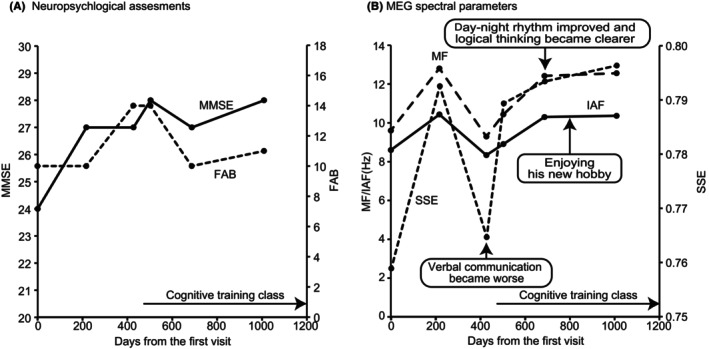
Changes in neuropsychological assessment scores and magnetoencephalography (MEG) spectral parameters in Case 1. (A) Neuropsychological assessment scores, (B) MEG spectral parameters. Boxes indicate clinical comments. MMSE, Mini Mental State Examination; FAB, Frontal Assessment Battery; MF, median frequency; IAF, individual alpha frequency; SSE, Shannon's spectral entropy.

A man in his 70s visited our memory clinic as recommended by his wife. He had retired 12 years earlier and enjoyed gardening as a hobby. Although he was unaware of any problems in his daily life, his wife had noticed that he had memory difficulties. On the same day (Day 0), he underwent a neuropsychological assessment and MEG scan, followed by a medical interview conducted by the physician in charge (author YH). MEG recordings and analyses were performed according to our previous studies.^3^, ^8^ Based on a comprehensive assessment, the patient was diagnosed with MCI. The physician identified the patient's day–night rhythm issues, noting that he had been staying awake until late, and advised him to go to bed and wake up early, as well as walk daily. However, the patient found it difficult to follow this advice considering that it contradicted the lifestyle habits developed from a young age; thus, he did not believe it to be harmful to his health at his current age. Follow‐up assessments (i.e., second examination) and accompanying interviews were arranged according to clinical requirements, and the patient was asked to return 6 months later. The second examination was performed on the Day 217. The MMSE score and three MEG parameters were increased, whereas the FAB score remained low (Figure 1 and Table 1). During the visit, the patient got lost in the hospital, suggesting difficulty with spatial orientation. Additionally, the clinical psychologist noticed that the patient had difficulty with verbal communication. Follow‐up interviews for the second examination were conducted on the Day 224, in which the physician advised him to get up early to re‐establish his day–night rhythm and asked him to return after 6 months. However, the patient was still not able to follow this advice.

**TABLE 1:** Neuropsychological assessment scores and MEG spectral parameters values at each examination.

	Examination	1	2	3	4	5	6
Case 1	Day	0	217	427	503	687	1009
	MMSE	24	27	27	28	27	28
	FAB	10	10	14	14	10	11
	MF	9.60	12.81	9.30	10.43	12.42	12.56
	IAF	8.60	10.43	8.34	8.90	10.30	10.36
	SSE	0.759	0.793	0.765	0.789	0.793	0.796
Case 2	Day	7	196	518	764		
	MMSE	28	27	22	28		
	FAB	12	9	13	12		
	MF	10.26	11.84	9.34	11.67		
	IAF	8.67	9.33	8.24	8.92		
	SSE	0.795	0.805	0.766	0.823		

Abbreviations: FAB, Frontal Assessment Battery; IAF, individual alpha frequency; MF, median frequency; MMSE, Mini Mental State Examination; SSE, Shannon's spectral entropy.

A third examination was performed on Day 427. The MMSE and FAB scores were increased, whereas all MEG parameters decreased (Figure 1 and Table 1). Although neuropsychological assessment scores were increased, the clinical psychologist noticed that the patient's difficulty with verbal communication had worsened, and the MEG parameters reflected the reports of the clinical psychologist. During the follow‐up interview for the third examination on Day 441, the physician identified limitations in the patient's ability to improve his cognitive status on his own and suggested that he participated in a weekly cognitive training class held in the hospital, starting on Day 448.

A pre‐class interview was conducted prior to class participation, in which each patient was individually interviewed every time by a physician (author YH or YS). During the interview, the patients' physical and psychological conditions were assessed and advice on well‐being provided. After the pre‐class interview, the patient actively participated in a 1‐h group work class organized by occupational therapists. The details of this class are described in our previous study.^8^ The patient has been actively participating in the classes and, according to medical records, has maintained an active lifestyle and enjoyed the sessions. However, his wife reported a worsening of his memory problems on the Day 497.

A fourth examination was performed on the Day 503. The clinical neuropsychologist's report indicated that the patient had difficulties in communication, although an occupational therapist mentioned that the patient's condition was well maintained at that time. Both the neuropsychological assessment scores and MEG parameters were improved (Figure 1 and Table 1), which were consistent with the perception of the occupational therapist. After the fourth examination, the patient was motivated to improve his cognition and maintain a more active lifestyle.

A fifth examination was performed on the Day 687. At that time, the patient's wife mentioned that his day–night rhythm had improved, and Dr. YS reported that his logical thinking had become clearer. However, the clinical psychologist reported difficulties in communication. The patient's neuropsychological assessment scores worsened (Figure 1 and Table 1), which was consistent with the neuropsychologist's report, whereas the MEG parameters improved, in line with the observations of his wife and physician. After the fifth examination, the patient discovered a new hobby, to which he devoted himself (medical notes on Day 763). This hobby was one he loved as a child but had abandoned for 40 years. Starting from that week, he spoke passionately about his hobby during the pre‐class interview, displaying great enthusiasm. It appeared that this newfound hobby significantly improved his quality of life.

A sixth examination was performed on the Day 1009. A clinical psychologist reported memory and attention difficulties in the patient, yet improved neuropsychological scores, which could be attributed to the practice effect (Figure 1 and Table 1). However, the practice effect alone could not explain the improved MEG spectral parameters. These increments possibly reflected the patient's fulfillment in daily life, particularly in relation to his newfound hobby.

Overall, changes in neuropsychological assessment scores and MEG spectral parameters showed similar trends after the patient joined the class (i.e., after the third examination), but there were discrepancies especially on the third examination: neurophysiological scores were increased, while MEG spectral parameters were not. The patient found it difficult to follow the doctor's advice until then, and his wife and medical staff did not report any improvement in his condition until the third examination. Overall, the changes in the MEG spectral parameters were consistent with the impressions of his wife and those of the medical staff.

### 2.2 Case 2

A woman in her 70s visited our memory clinic (Day 0) with subjective complaints of misplacing items and experiencing difficulties in calculations. Her husband, whom she had taken care of, had been suffering from dementia for the past 20 years. Unfortunately, he passed away the previous year, and, since then, the patient had been struggling with depression. Although she frequently ate out and traveled with her friends and sister before, she refused to meet them after her husband's demise.

During the initial interview, the physician recommended her to spend more time with her friends and consume a protein‐rich diet, as her diet was unbalanced due to her suffering from diabetes mellitus. She underwent the first examination on the Day 7 and was diagnosed with MCI by Dr. YH, based on a comprehensive judgment at the follow‐up interview on Day 28. She was advised to maintain a healthy day–night rhythm, keep a diary, and regularly go for a walk. Additionally, she was advised to participate in a weekly cognitive training class starting from Day 42. According to the medical notes, she reported improvements in her memory (Day 91), started keeping a diary (Day 141), stopped napping, and started walking regularly (Day 154). She expressed a desire to travel again with her friends on Day 175. Despite her active appearance, her condition was unstable because she often became sick and suffered from dizziness during bad weather, especially during seasonal changes.

**FIGURE 2:**
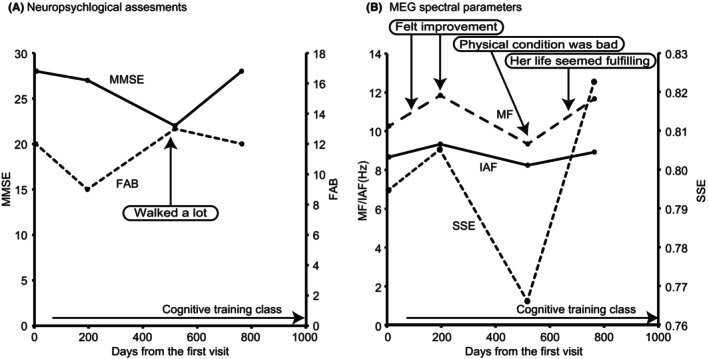
Changes in neuropsychological assessment scores and magnetoencephalography (MEG) spectral parameters in Case 2. (A) Neuropsychological assessment scores, (B) MEG spectral parameters. MMSE, Mini Mental State Examination; FAB, Frontal Assessment Battery; MF, Median Frequency; IAF, Individual Alpha Frequency; SSE, Shannon's spectral entropy.

A second examination was performed on the Day 196. Her physical condition was poor due to the bad weather, which was reflected in the lower MMSE and FAB scores (Figure 2 and Table 1). However, she perceived a performance improvement during the neuropsychological assessment. MEG spectral parameters increased, which was consistent with her subjective experience, but not the changes in neuropsychological assessment scores.

A third examination was performed on the Day 518. During the transition from summer to autumn, her condition was poor due to the weather on that day, although she was getting more active and had walked a lot a few weeks before. The lowest scores and values in the MMSE and three MEG spectral parameters reflected her poor condition on that day, although a high FAB score indicated her active lifestyle (Figure 2 and Table 1). The patient became more active after the third examination. She traveled several times with her sister and started visiting a nursing care center to increase her opportunities for social interactions and receive physical and mental exercise. Although she reported that her memory had worsened (Day 644), her caregiver mentioned that she often went out actively, and her life seemed more fulfilling than before (Day 665).

A fourth examination was performed on Day 746, during mid‐spring. She experienced dizziness a few days before the examination and was unable to walk daily due to bad weather. Fortunately, her physical condition on the day of the examination was good. A clinical psychologist reported that she actively and happily participated in assessments. The MMSE and FAB scores were the same as those at the first assessment, whereas the MF and IAF were higher than those at the first assessment, and the SSE was the highest (Figure 2 and Table 1).

Overall, her condition was well‐maintained, with some fluctuations. Although changes in MEG spectral parameters and MMSE scores showed similar trends with minor differences, changes in MEG spectral parameters were consistent with the subjective reports from her caregiver and medical staff.

## 3 DISCUSSION

We retrospectively analyzed the clinical records of two patients with MCI who participated in our weekly cognitive training classes without pharmacological treatment to improve their cognition. After a few visits, both patients became more active and enjoyed their lives. Changes in longitudinal data of neuropsychological assessments (MMSE and FAB) and MEG spectral parameters showed similar trends, with some discrepancies. In particular, the changes in MEG spectral parameters were more consistent with the subjective reports from caregivers and medical staff.

Non‐pharmacological treatments are often effective in improving the condition of patients with cognitive impairment.^1^, ^2^ To provide better treatment, monitoring outcomes repeatedly and regularly is important. Cognitive status is usually evaluated using neuropsychological assessments; however, these assessments have inherent limitations, such as the practice effect. Such effect poses a particular problem in longitudinal studies,^4^, ^5^ because patients become accustomed to and can prepare for assessments.

In the present study, the patient from Case 1 showed a continuous improvement in MMSE scores. However, this did not correspond with his wife's and medical staff's observations. Moreover, the patient was not able to follow the doctor's advice to change his lifestyle until the third examination; therefore, it was unlikely that his condition had stably improved. The MEG spectral parameters were similar in the first and third examinations, matching our clinical observations. Constant improvements in the MMSE and FAB scores could be partially explained by the practice effect.^4^, ^5^

The patient from Case 2 exhibited the opposite trend. She changed her lifestyle after the first visit and her condition was subjectively improved. This was consistent with the changes in MEG spectral parameters, which increased between the first and second examinations. However, the MMSE and FAB scores were decreased. Unfortunately, we could not provide any explanation for these decrements. Except for the changes between the first and second examinations, the changes in the MMSE score and MEG spectral parameters showed comparable trends. However, the change in FAB scores presented a distinctive trend. We speculated that this difference could be explained by the contributions of regions outside the cortex. The FAB was designed to assess frontal function (e.g., executive function), and parts of the assessment are associated with motor control tasks.^9^ Motor performance depends on activity not only in the frontal cortex but also in other brain regions, such as the basal ganglia and cerebellum,^10^ which contribute to unconscious motor control.^11^, ^12^ Hence, these regions could have different characteristics in terms of practice effects from that of the cortex, leading to different trends in FAB.

The present study has four potential limitations; first, the patients from both cases presented MCI with relatively preserved cognition. The comparable trends observed between MEG spectral parameters and MMSE scores may, therefore, be applicable only to similar cases. Future studies should investigate the relationship between clinical conditions and MEG spectral parameters in patients with dementia due to various pathologies. Second, we only reported the MMSE and FAB scores because previous studies reported that they correlate with MEG spectral parameters.^3^ However, some important neuropsychological assessment scores, such as the Alzheimer's Disease Assessment Scale, were not included. Future group studies are required to clarify their relationships with patients' conditions and MEG spectral parameters. Third, as a limitation of the case study format, only two cases were described without control groups. Neither statistical analysis nor comparison with control data was applied. We anticipate that future studies with different cases and group data should address these points and improve the reliability of the present monitoring method. Fourth, because this was an observational study, we could not identify the cause of improvements in the patients' conditions. Although weekly cognitive training as the main cause is plausible, other factors, such as the pre‐class interview, may have affected their conditions. We also expect that future studies address this question.

Taken together, these two cases suggest that changes in MEG spectral parameters reflect the changes in clinical conditions of patients with MCI better than those in neuropsychological assessment scores, possibly because MEG spectral parameters are affected by the practice effect much less than neuropsychological assessments. MEG is a non‐invasive, patient‐friendly neuroimaging technique that uses no radiation or injections for scanning and is completed in 10 minutes, including preparations. Therefore, MEG is a promising tool for monitoring treatment outcomes in patients with MCI.

## AUTHOR CONTRIBUTIONS

**Yoko Hirata:** Conceptualization; supervision. **Hideyuki Hoshi:** Conceptualization; writing – review and editing. **Momoko kobayashi:** Data curation. **Keita Shibamiya:** Data curation. **Keisuke Fukasawa:** Data curation. **Sayuri Ichikawa:** Data curation. **Yoshihito Shigihara:** Conceptualization; data curation; formal analysis; funding acquisition; investigation; methodology; project administration; resources; software; supervision; validation; visualization; writing – original draft; writing – review and editing.

## FUNDING INFORMATION

This study was partially funded by RICOH Co., Ltd. (Japan). The funder had no role in the study concept/design, methods, data collection and analysis, decision to publish, or preparation of the manuscript. Financial support was provided to cover English language editing and publication fees.

## CONFLICT OF INTEREST STATEMENT

Dr. Yoshihito Shigihara is leading a joint research project with RICOH Co., Ltd. (Japan, manufacturer of magnetoencephalography equipment). Mr. Hideyuki Hoshi is now employed by RICOH Co., Ltd. Dr. Yoko Hirata is partially sponsored for attending academic conferences by RICOH Co., Ltd. Momoko Kobayashi, Keita Shibamiya, Keisuke Fukasawa, and Sayuri Ichikawa have no conflicts of interest to declare.

## ETHICS STATEMENT

This study was conducted in accordance with the Declaration of Helsinki and relevant Japanese regulations and approved by the Ethics Committee of the Kumagaya General Hospital (approval number: #0025).

## CONSENT

Written informed consent for research use and publication was obtained from both patients and their caregivers.

## Data Availability

All the data generated or analyzed during this study are included in this article. Further enquiries can be directed to the corresponding author.
